# Cardiopulmonary Exercise Testing in Heart Failure

**DOI:** 10.3390/jcdd11030070

**Published:** 2024-02-20

**Authors:** Michel Juarez, Cristian Castillo-Rodriguez, Dina Soliman, Gaspar Del Rio-Pertuz, Kenneth Nugent

**Affiliations:** 1 Department of Internal Medicine, Texas Tech University Health Sciences Center, Lubbock, TX 79430, USA; michel.juarez@ttuhsc.edu (M.J.); cristian.castillo-rodriguez@ttuhsc.edu (C.C.-R.); dina.soliman@ttuhsc.edu (D.S.); 2 Division of Cardiology, Department of Internal Medicine, University of Minnesota, Minneapolis, MN 55455, USA; delri031@umn.edu

**Keywords:** heart failure, cardiopulmonary exercise testing, prognosis, treatment

## Abstract

Cardiopulmonary exercise testing (CPET) provides important information for the assessment and management of patients with heart failure. This testing measures the respiratory and cardiac responses to exercise and allows measurement of the oxygen uptake ($\dot{V}$O_2_) max and the relationship between minute ventilation ($\dot{V}$E) and carbon dioxide excretion ($\dot{V}$CO_2_). These two parameters help classify patients into categories that help predict prognosis, and patients with a $\dot{V}$O_2_ < 14 mL/kg/min and $\dot{V}$E/$\dot{V}$CO_2_ slope >35 have a poor prognosis. This testing has been used in drug trials to determine complex physiologic responses to medications, such as angiotensin-converting enzyme inhibitors. For example, a study with enalapril demonstrated that the peak $\dot{V}$O_2_ was 14.6 ± 1.6 mL/kg/min on placebo and 15.8 ± 2.0 mL/kg/min on enalapril after 15 days of treatment. The $\dot{V}$E/$\dot{V}$CO_2_ slopes were 43 ± 8 on placebo and 39 ± 7 on enalapril. Chronic heart failure and reduced physical activity measured by cardiopulmonary exercise testing are associated with increases in BNP, and several studies have demonstrated that cardiac rehabilitation is associated with reductions in BNP and increases in $\dot{V}$O_2_. Therefore, BNP measurements can help determine the benefits of cardiac rehabilitation and provide indirect estimates of changes in $\dot{V}$O_2_. In addition, measurement of microRNAs can determine the status of skeletal muscle used during physical activity and the changes associated with rehabilitation. However, CPET requires complicated technology, and simpler methods to measure physical activity could help clinicians to manage their patients. Recent advances in technology have led to the development of portable cardiopulmonary exercise testing equipment, which can be used in various routine physical activities, such as walking upstairs, sweeping the floor, and making the bed, to provide patients and clinicians a better understanding of the patient’s current symptoms. Finally, current smart watches can provide important information about the cardiorespiratory system, identify unexpected clinical problems, and help monitor the response to treatment. The organized use of these devices could contribute to the management of certain aspects of these patients’ care, such as monitoring the treatment of atrial fibrillation. This review article provides a comprehensive overview of the current use of CPET in heart failure patients and discusses exercise principles, methods, clinical applications, and prognostic implications.

## 1. Introduction

Heart failure (HF) is a clinical disorder in which the heart cannot pump blood to the body at a rate commensurate with its metabolic requirements or can do so only by using high filling pressures. As the prevalence of HF continues to increase globally, there is a growing need for precise diagnostic and prognostic tools to guide therapeutic strategies to improve patient outcomes and to provide the basis for clinical trials. Cardiopulmonary exercise testing (CPET) is a valuable and versatile tool used in the classification of HF; these tests characterize the dynamic interactions between the cardiovascular, respiratory, and metabolic systems during physical exertion [1]. This testing involves the systematic measurement of respiratory gases, heart rate, and other physiological parameters during controlled exercise. This non-invasive assessment provides a comprehensive evaluation of exercise capacity and helps identify abnormalities in cardiopulmonary function that may not be apparent at rest [2]. In the context of HF, CPET offers a unique perspective, allowing for the classification of patients based on their functional capacity, exercise tolerance, and response to exertion [3].

The classification of HF traditionally relies on clinical parameters, such as symptoms, left ventricular ejection fraction, and structural abnormalities. However, these measures do not describe the dynamic nature of this disorder during physical activity. In contrast, CPET provides a dynamic and objective assessment of exercise capacity and provides information about the severity of impairment in the cardiovascular and respiratory systems. This information is particularly valuable in distinguishing between different stages of HF and targeting therapeutic interventions to individual patient needs [4].

This review will discuss the physiologic responses during CPET, its role in assessing exercise capacity, and its significance in the classification of HF and the evaluation of drugs used in HF patients. By considering the complex cardiopulmonary responses to exercise, this review will highlight the important information derived from CPET studies in the characterization and understanding of HF, which could lead to more precise and personalized management strategies.

## 2. Background of Cardiopulmonary Exercise Testing

Cardiopulmonary exercise testing is a diagnostic tool used to measure cardiovascular and pulmonary function during exercise. It provides information about an individual’s exercise capacity, fitness, cardiovascular function, and pulmonary function. By interpreting its parameters, it is possible to evaluate and diagnose cardiovascular diseases, pulmonary disorders, and metabolic abnormalities. Dyspnea and fatigue are symptoms that could be present in cardiovascular conditions, such as HF; however, other pulmonary conditions and even deconditioning can present with dyspnea and fatigue. Cardiopulmonary testing is a valuable tool that can help in the diagnosis and in medical decisions needed in the management of patients with HF [5,6].

### Testing Protocols in Heart Failure

Exercise tolerance is usually determined with a treadmill or cycle ergometer; CPET is a more comprehensive and specialized type of exercise testing that provides an accurate and objective measure of cardiorespiratory function. In general, a CPET report has four divisions: the first part, called metabolics, includes oxygen uptake ($\dot{V}$O_2_), carbon dioxide excretion ($\dot{V}$CO_2_), and respiratory exchange ratio (RER); the second part, called cardiac, includes heart rate and systolic and diastolic blood pressure; the third part, called ventilation, includes minute ventilation, respiratory rate, and dead space ventilation; the fourth part, called gas exchange, includes FiO_2_, SpO_2_, pH, PaCO_2_, PaO_2_, arterial–alveolar oxygen difference pressure, and lactate levels [7,8]. Therefore, CPET provides invaluable diagnostic and prognostic information about clinical disorders associated with exercise intolerance. 

Cardiopulmonary exercise testing can be performed using either incremental or constant work rate protocols, defined by whether the work rate progressively increases or remains constant during the test. The main objective of incremental tests is to maximally stress O_2_ transport and uptake, and these tests are often used in clinical medicine. Ramp protocols are preferred over conventional incremental tests whenever possible. Several ramp grades are commonly used in patients, and these include 5 Watts/min, 7 Watts/min, 10 Watts/min, and 15 Watts/min. The choice of ramp protocol steepness should be based on the patient’s expected exercise tolerance, and the test should last between 8 and 12 min. Two advantages of ramp protocols are important considerations: first, the work rate increase does not have a brisk step increase used in step protocols (e.g., 25 Watts every 3 min); second, the trends in parameters during the test are not affected by protocol steps, making physiological responses linear and easier to interpret [8,9,10,11,12].

While standard exercise tests (ET) have long been considered the initial test used before more expensive and invasive procedures, such as angiography, bypass surgery, transplantation, and device implantation, gas exchange measurements during exercise can improve decision making. Cardiopulmonary exercise testing is a specialized type of ET that provides a more accurate and objective measure of cardiopulmonary function.

Patients with HF with both preserved and reduced ejection fractions have changes in their cardiovascular, respiratory, muscular, sympathetic, and neurohormonal systems [7]. Cardiopulmonary exercise testing cannot consistently determine the correct classification of patients with HF. Studies have shown that CPET variables are equally abnormal in both groups of patients when they are matched by clinical characteristics, and a reduced $\dot{V}$O_2_ predicts outcomes in both groups of patients. Other factors, such as gender, height, weight, and age, also affect gas exchange and need consideration when analyzing CPET results [7,13,14].

## 3. Cardiovascular and Respiratory Responses to Exercise

During prolonged exercise, there is an increase in the oxygen requirement to meet metabolic demands. To provide more oxygen to the peripheral tissue during exercise, the cardiovascular system adapts by increasing systolic blood pressure, reducing systemic vascular resistance, increasing blood supply to the muscles, and increasing venous return to the heart, facilitated by the squeezing effect of the calf muscles. Cardiac output increases because of increases in both heart rate (HR) and stroke volume (SV). At the same time, there is an increase in minute ventilation in response to exercise with increases in both tidal volume (*V*_T_) and respiratory rate, driven by carbon dioxide (CO_2_) production. Any limitation of these physiological cardiovascular or respiratory responses will eventually cause exercise intolerance and an overall decrease in exercise capacity. The goal of CPET is to diagnose and localize the cause of exercise limitation using non-invasive dynamic methods [15].

**Figure 1:**
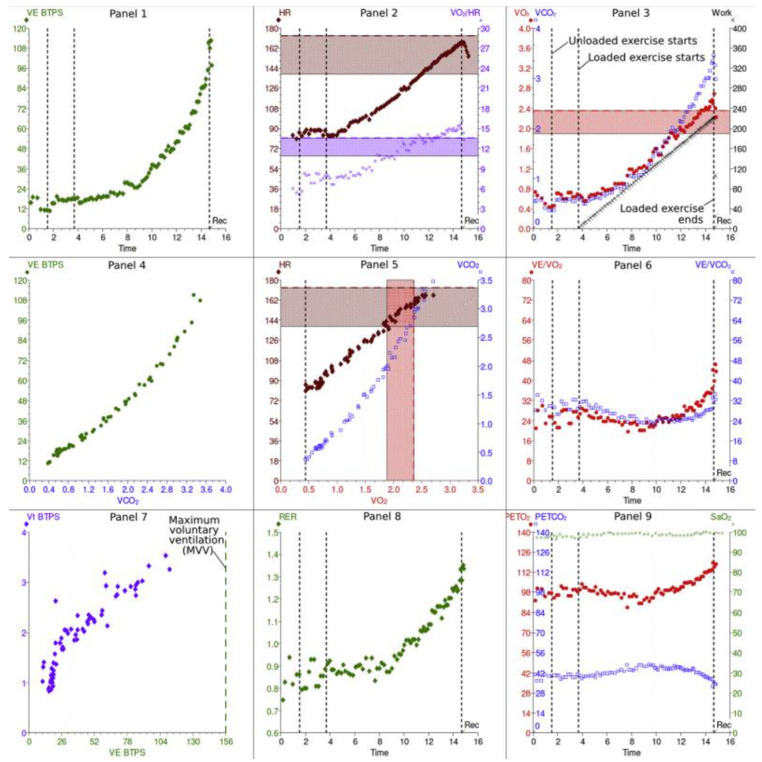
Cardiopulmonary exercise results: 9 panels.

The results of CPET can be illustrated with a nine-plot panel (Figure 1). In this article, these panels are classified according to the function they reflect in either cardiovascular, gas exchange, or ventilatory response panels.

These panels illustrate the information collected during cardiopulmonary exercise testing. Please see the text for more discussion of the information in each panel. This figure is reproduced from Chambers and Wisely, based on the STM Permissions Guidelines in which Elsevier participates. Chambers D, Wisely N. Cardiopulmonary exercise testing—a beginner’s guide to the nine-panel plot [15].

Abbreviations: Panel 2, 5: HR—heart rate; SV—stroke volume; Panel 7: VT—tidal volume; CO_2_—carbon dioxide; Panel 2, 3, 6: $\dot{V}$O_2_—oxygen uptake; Panel 3, 5, 6: $\dot{V}$CO_2_—CO_2_ production; Panel 3: WR—work rate; AT—anaerobic threshold; Panel 1, 4: $\dot{V}$E—minute ventilation; V/Q—ventilation/perfusion ratio; EqO_2_—oxygen ventilatory equivalent; EqCO_2_—CO_2_ ventilatory equivalent; Panel 9: P_ET_O_2_—end-tidal pressure of O_2_; P_ET_CO_2_—end-tidal pressure of CO_2_; Panel 7: MVV—maximum voluntary ventilation; FEV_1_—forced expiratory volume in 1 s; Panel 8: RER—respiratory exchange ratio; BF—breathing frequency.

### 3.1. Cardiovascular Panels

Panel 3: O_2_ uptake ($\dot{V}$O_2_) and CO_2_ output ($\dot{V}$CO_2_) vs. time plus relationship of peak $\dot{V}$O_2_ and work rate (WR).

Peak $\dot{V}$O_2_ indicates peak exercise capacity and oxygen uptake at the end of an incremental exercise test; it depends on patient effort. An increase in Δ$\dot{V}$O_2_/ΔWR provides information about aerobic exercise capacity. Under normal conditions, a person should achieve more than 80% of the predicted work measured in Watts and is expected to have a linear increase in the $\dot{V}$O_2_/WR at a rate of 10 mL/min per Watt [15,16,17]. During exercise, a lower ratio suggests less oxygen delivery and increased anaerobic metabolism. This can happen with peripheral artery, cardiovascular, pulmonary vascular, and lung diseases [18,19].

Panel 2: Heart rate and oxygen pulse in relation to time. The O_2_ pulse ($\dot{V}$O_2_/HR) represents the volume of oxygen extracted by the tissues per heartbeat. The maximum heart rate during exercise can be estimated with this equation: predicted maximum = 220 beats per min—age [15]. A healthy person should be able to achieve more than 85% of the maximum calculated HR. In patients who are not taking beta-blocking medications, this reflects the chronotropic competence of the heart in response to exercise [18].

Panel 5: Relationship of CO_2_ output and O_2_ uptake and the relationship between HR and $\dot{V}$O_2_. The key variable determined in this panel is the anaerobic threshold (AT) during exercise, which is the point at which the O_2_ demand of the muscles surpasses the cardiopulmonary system’s O_2_ supply capacity. Consequently, skeletal muscle cells generate ATP through anaerobic metabolism, producing lactic acid, which is buffered by bicarbonates, generating excess CO_2_. The AT corresponds to the point at which $\dot{V}$CO_2_ increases disproportionately when compared with $\dot{V}$O_2_ in Panel 5, or the point at which $\dot{V}$E/$\dot{V}$O_2_ starts to increase, whereas $\dot{V}$E/$\dot{V}$CO_2_ stays comparatively constant, as shown in Panel 6 [15].

### 3.2. Pulmonary Gas Exchange Panels

Panel 6: The minute ventilation ($\dot{V}$E) vs. $\dot{V}$O_2_ and vs. $\dot{V}$CO_2_ (ventilatory equivalents). Adequate gas exchange is measured by the ventilatory equivalents: EqO_2_ ≈ $\dot{V}$E/$\dot{V}$O_2_ and EqCO_2_ ≈ $\dot{V}$E/$\dot{V}$CO_2_. In other words, this measures how many liters of minute ventilation are needed to take up 1 L of O_2_ or exhale 1 L of CO_2_. The lower these ratios are, the better the gas exchange and breathing effort are, and vice versa [17].

Panel 4: The relationship of $\dot{V}$E and $\dot{V}$CO_2_. The $\dot{V}$E/$\dot{V}$CO_2_ slope is another key variable of CPET, because it predicts cardiac-related morbidity and mortality in patients with congestive HF and is an independent predictor of perioperative morbidity post-cardiac transplant surgeries [19,20].

Panel 9: End-tidal pressures of O_2_ (P_ET_O_2_) and CO_2_ (P_ET_CO_2_) vs. time reflect pulmonary gas exchange and ventilation/perfusion mismatch. The more efficient the ventilation, the higher the P_ET_CO_2_ and the lower the P_ET_O_2_ in normal lungs. Of note, P_ET_CO_2_ is greater than PaCO_2_ during exercise and lower than PaCO_2_ at rest [17]. Decreased end-tidal CO_2_ during exercise indicates hyperventilation or ventilation/perfusion mismatch; however, increased P_ET_CO_2_ can also represent hypoventilation of the alveoli caused by either obstructive or restrictive lung disorders [17].

### 3.3. Ventilatory Response Panels

Panel 1: Relationship between $\dot{V}$E and WR vs. time. The maximum voluntary ventilation (MVV) is calculated indirectly as forced expiratory volume in 1 s (FEV_1_) × 40 or can be determined by direct measurement of MVV. Exercise is usually not limited by respiratory function but rather by heart rate in normal subjects.

Panel 8: Respiratory exchange rate (RER). The RER describes the ratio of CO_2_ production to O_2_ uptake ($\dot{V}$CO_2_/$\dot{V}$O_2_); the normal ratio at rest should be 0.7 to 1. The RER is considered a key variable in patients with chronic HF because higher RERs are associated with worse outcomes, including all-cause mortality and hospital re-admission due to HF exacerbations. The RER is also a prognostic predictor of an AT point, which is helpful in patients who cannot achieve peak $\dot{V}$O_2_ because of either musculoskeletal, cardiovascular, or lung diseases [21].

Panel 7: Breathing pattern. Relationships of tidal volume (V_T_), minute ventilation ($\dot{V}$E), and breathing frequency (BF). In obstructive lung diseases, Vt increases, and BF is limited due to prolonged expiratory time. This pattern results in slow, deep breathing. However, in restrictive lung diseases, breathing is shallow and fast because the Vt is limited, secondary to reduced lung volumes, but the BF increases [17].

## 4. Exercise Parameters of Increased Importance in CHF Patients

Cardiac output is usually normal at rest in patients with mild HF, but it does not increase with physical activity [22]. The peak $\dot{V}$O_2_ in HF is directly related to peak cardiac output and perfusion of exercising muscles. The failure to increase cardiac output decreases perfusion to these muscles and can cause anaerobic metabolism at lower workloads and muscle fatigue. These patients often do not reach a true $\dot{V}$O_2_ max, and their $\dot{V}$O_2_ at the end of exercise is called “peak $\dot{V}$O_2_”.

Cardiopulmonary exercise testing provides an important and objective method for determining the functional capacity of patients with CHF. There are several variables in addition to $\dot{V}$O_2_ that have prognostic implications in HF that can only be measured using the CPET. During CPET, peak $\dot{V}$O_2_ and the $\dot{V}$E/$\dot{V}$CO_2_ slope during exercise are important parameters used to measure functional capacity and have prognostic value. Exercise capacity, measured by peak $\dot{V}$O_2_, is an excellent prognostic index in patients with CHF. In addition, several studies have demonstrated that the $\dot{V}$E/$\dot{V}$CO_2_ slope has an equivalent or even superior prognostic value compared to the measurement of peak $\dot{V}$O_2_ in patients with CHF [23,24,25]. Other important predictors in HF patients are discussed in the next paragraphs.

### 4.1. $\dot{V}$E/CO_2_ Slope

In chronic HF, minute ventilation ($\dot{V}$E) for a given level of carbon dioxide production ($\dot{V}$CO_2_) might be abnormally high during exercise due to increased dead space ventilation, decreased lung compliance, increased chemo- and metabolic reflex sensitivity, early metabolic acidosis, and abnormal pulmonary hemodynamics. The relationship between $\dot{V}$E and $\dot{V}$CO_2_, in L/min, represents the matching of ventilation and perfusion within the pulmonary system, and a ratio < 30 is considered normal. The explanation for this analysis is that higher ratios indicate that a higher minute ventilation is needed to excrete CO_2_, which reflects inefficiency in the system. The $\dot{V}$E/$\dot{V}$CO_2_ slope is a continuous variable that predicted major cardiac events in a study with 448 patients with chronic HF (both HFrEF and HFpEF); a very poor prognosis occurred in patients with a slope ≥45 [26]. Several studies have shown a direct correlation between the slope and major adverse cardiac events (MACE), mortality, transplantation, or left ventricular assist device implantation. Studies have also predicted a cutoff for hospitalization >32.9. 

There are four levels of ventilatory classification (VC) in HF:

VC-I < 30—negligible risk of MACE
VC-II 30–35.9—low risk of MACE
VC-III 36–44.9—moderate risk of MACE
VC-IV ≥ 45—high risk of MACE

### 4.2. Peak $\dot{V}$O_2_

This parameter is considered as a universal prognostic marker. The $\dot{V}$O_2_ is the amount of oxygen being used by the tissues per minute, and the peak $\dot{V}$O_2_ represents the $\dot{V}$O_2_ at the peak of exercise. There are three measured units in CPET reports: the absolute peak $\dot{V}$O_2_ is measured in mL O_2_/min, the relative peak $\dot{V}$O_2_ in mL O_2_/kg/min/, or the predicted peak $\dot{V}$O_2_ in %, based on the study by Neder et al., which used anthropometric characteristics to create predicted peak $\dot{V}$O_2_ tables [27].

Patients with a peak $\dot{V}$O_2_ ≤ 10 mL/kg/min have the worst prognosis (normal value with exercise is above 20 mL/kg/min). In patients with HF, the percentage of predicted peak $\dot{V}$O_2_ is normal if ≥100%, and a percentage of predicted $\dot{V}$O_2_ < 50% predicts a poor prognosis.

### 4.3. Respiratory Equivalent Ratio (RER) and Anaerobic Threshold

Defined as the $\dot{V}$CO_2_/$\dot{V}$O_2_ ratio, a normal RER value is usually between 0.7 and 1.0, with a low level (0.7) indicating fat metabolism and a high level (1.0) indicating carbohydrate metabolism, and ratios in between indicating mixed fuel consumption. As exercise increases to higher intensities, $\dot{V}$CO_2_ exceeds $\dot{V}$O_2_, increasing the ratio. Currently, this ratio is the best non-invasive indicator of exercise effort, and a peak value ≥1.10 is considered an excellent exercise effort [28].

The principle behind this analysis is based on the classic pathways of aerobic and anaerobic glycolysis in which the CO_2_ produced during the anaerobic glycolysis is higher than in aerobic metabolism, reflecting a less-efficient system of producing energy. The anaerobic threshold represents the point of exercise at which the $\dot{V}$CO_2_ increases exponentially relative to the $\dot{V}$O_2_. However, this parameter is not specific because lung disease, anemia, myopathies, and general deconditioning can affect the threshold.

### 4.4. Partial Pressure of End-Tidal Carbon Dioxide (PETCO_2_) in mmHg at Rest and during Exercise

The end-tidal carbon dioxide level reflects the matching of ventilation and perfusion in the pulmonary and cardiac systems. Therefore, it reflects disease severity in a number of clinical disorders, including HF, pulmonary hypertension, COPD, and interstitial lung disease.

### 4.5. Exercise Oscillatory Ventilation

Periodic breathing is a form of irregular breathing characterized by a regular cyclic variation in ventilation with increases and decreases in tidal volume interrupted by pauses with a cycle length of approximately 1 min. Periodic breathing, or Cheyne and Stokes respiration in the resting state, has been recognized as a characteristic of HF for over 200 years. This breathing pattern can also occur during exercise and is called exercise oscillatory ventilation (EOV). This develops in a significant percentage of HF patients [28]. There is no standard definition currently available; the most commonly accepted one is an oscillatory pattern at rest that persists for ≥60% of the exercise test at an amplitude of ≥15% of the average resting value. Ventilation without EOV during exercise predicts event-free survival in HF patients. However, periodic breathing reflects advanced disease severity and predicts a poor prognosis in patients with HF whether at rest, during sleep, or during exercise.

## 5. Protocols and Graded Exercise Testing

Different exercise testing protocols have been described in the literature. This equipment, including both cycle ergometers and treadmills, can measure the $\dot{V}$O_2_ max, $\dot{V}$CO_2_, and $\dot{V}$E. Generic types of protocols include progressive, incremental, and multistage (every 3 min, with a “pseudo” steady-state metabolic level at each stage) protocols [29]. In clinical practice, maximal incremental protocols are often used. Changes in $\dot{V}$O_2_, $\dot{V}$CO_2_, and $\dot{V}$E often lag behind changes in work rate. Therefore, the preferred protocol should increase the work rate at a constant rate.

### 5.1. Important Exercise Protocol Characteristics

Exercise time should be maintained between 6 and 12 min, which is probably the optimal time to obtain efficient and useful metabolic and functional information. Both ergometer and treadmill protocols should be preceded by an initial warm-up phase at 0 Watts for 1–3 min and should include a recovery phase of at least 5 min with a tapering workload.

### 5.2. Gas Analysis Systems

Respiratory gas exchange measurement is a crucial part of the CPET technique; maximal oxygen uptake and anaerobic threshold determination serve as indices of exercise capacity that can be applied to various clinical problems, such as differential diagnosis of exertional dyspnea and fatigue, in which maximal oxygen uptake and anaerobic threshold determination are combined with a simultaneous assessment of circulatory and ventilatory reserves. Arterial blood gas determination can be used to define ventilation–perfusion relationships. During exercise, both components of the Fick equation (cardiac output [CO] and arteriovenous O_2_ difference [(A-V) O_2_]) should increase; the $\dot{V}$O_2_ max is the maximum amount of O_2_ that can be used for metabolic work for a given form of exercise and is related to the maximum CO and (A-V) O_2_ that can be attained [30].

## 6. Interpretation of CPET Parameters

Important parameters include the electrocardiogram, blood pressure, heart rate (HR), peripheral oxygen saturation (SpO_2_), oxygen consumption ($\dot{V}$O_2_), carbon dioxide production ($\dot{V}$CO_2_), and ventilation ($\dot{V}$E) [17].

### 6.1. Target Exercise Load for CPET

The most important benefit of the use of a bike ergometer instead of a treadmill is the linear increase in workload. With a treadmill, it is possible to change slope and speed, but not the workload itself, and therefore it is almost impossible to generate a linear increase in workload that provides an accurate objective measurement of the $\dot{V}$O_2_/work rate relationship. The exercise ramp protocol should have an exercise duration of ~10 min with an interval ranging from 8 to 12 min [31]. This means that the workload should be adjusted for each patient to achieve this test duration. This is easier with a ramp exercise protocol on a bike ergometer on which the load can be easily changed. This task is more difficult and less standardized with treadmill protocols.

### 6.2. Exercise Limits in Uncomplicated Medical Disorders

Cardiac disorders: Patients with cardiac disorders usually meet their exercise limit when they reach their predicted maximum heart rate. This typically occurs at a lower work level and lower $\dot{V}$O_2_ max. They often cross an anaerobic threshold at a lower work rate, and they have higher $\dot{V}$E/$\dot{V}$CO_2_ slopes.

Pulmonary disorders: These patients reach their maximum exercise capacity when they reach their ventilatory limit. The respiratory pattern includes higher respiratory rates and lower tidal volumes that do not increase during exercise. They may desaturate during exercise.

Muscular disorders: These patients often stop exercise because of muscle fatigue and have not reached either a cardiac or respiratory limit. Depending on the exact metabolic abnormality in these muscles, these patients may or may not have increased anaerobic metabolism. Some muscular disorders also involve cardiac muscle, and this makes this interpretation more difficult [32,33]. In addition, patients with HF also lose muscle mass, which contributes to their exercise limitation.

However, patients with HF frequently have more than one medical disorder, and this additional comorbidity can contribute to both symptoms, physical limitations, and disease progression [34]. In particular, anemia, pulmonary disorders such as sleep apnea, and skeletal muscle weakness/wasting contribute to these patients’ physical impairment and disability.

## 7. Risk Stratification in HF Using CPET

The measurement of several variables improves prognostic accuracy and prediction relationships. In general, a peak $\dot{V}$O_2_ ≥ 20 mL/kg/min, a $\dot{V}$E/$\dot{V}$CO_2_ slope <30, no exercise oscillatory ventilation, and a $\dot{V}$O_2_ anaerobic threshold >11 mL/kg/min predicts an excellent prognosis during the next 1–4 years, with few major adverse events (≥90% event free); therefore, these patients can be retested in 4 years following appropriate medical management of their HF [35]. However, patients with a peak $\dot{V}$O_2_ < 10 mL/kg/min, $\dot{V}$E/$\dot{V}$CO_2_ slope ≥36, presence of exercise oscillatory ventilation, and $\dot{V}$O_2_ anaerobic threshold <11 mL/kg/min have a very poor prognosis, with greater than 20% mortality in 1 year.

### 7.1. Prognostic Value of CPET Parameters

Careful measurement of ventilation and O2 uptake patterns in HF can quantify disease severity and prognosis. Cardiopulmonary exercise testing is routinely used in the prognostic evaluation of patients with HF with reduced ejection fractions (HFrEF), especially when advanced therapies are considered. The first study evaluating the prognostic utility of CPET parameters dates back to 1991, when Mancini et al. published their groundbreaking work on the predictive value of peak $\dot{V}$O_2_, and established a peak $\dot{V}$O_2_ cutoff of <14 mL/kg/min as a criterion for which 1-year survival was significantly lower (47%) than survival with transplantation (70%) [36]. In contrast, individuals with a peak $\dot{V}$O_2_ > 14 mL/kg/min had a higher (95%) 1-year survival, suggesting that cardiac transplantation could be safely deferred in ambulatory patients with severe left ventricular dysfunction and a peak exercise $\dot{V}$O_2_ of more than 14 mL/kg/min. Following these results, studies have focused on ventilatory efficiency during CPET, expressed as the minute ventilation per carbon dioxide production ($\dot{V}$E/$\dot{V}$CO_2_) slope, and have demonstrated that it is a robust prognostic marker in patients with HFrEF [37,38,39,40]. A $\dot{V}$E/$\dot{V}$CO_2_ slope >34 to 36 identifies high-risk HF patients and provides prognostic information above and beyond peak $\dot{V}$O_2_ [25].

Initial studies have focused on the prognostic utility of CPET parameters in patients with HFrEF, demonstrating that the prognostic value of peak $\dot{V}$O_2_ and the $\dot{V}$E/$\dot{V}$CO_2_ slope in patients with HFrEF is powerful and well-established [28,37]. There is a now an interest in studying CPET in patients with HFpEF and HFmrEF, since many studies have reported that the predictive value of peak $\dot{V}$O_2_ and $\dot{V}$E/$\dot{V}$CO_2_ in patients with LVEF > 40% can be as good as in patients with severe left ventricular dysfunction [41,42,43,44,45]. However, these data have conflicting results.

### 7.2. Risk Stratification in Heart Failure

Risk predictions of hospitalization and/or death is important for both patients and physicians. For patients, this information can help them understand the severity of their illness and thereby guide patients in decision making, including preferences for advanced HF therapies and/or end-of-life care. For physicians, these predictions provide guidance toward which treatment to offer patients, and when to offer treatment. Interpretation of cardiopulmonary exercise testing in the individual patient should start with an assessment of whether maximal effort was achieved or not, as indicated by RER [38]. A RER >1.0 to 1.1 indicates maximal patient physiological effort. A heart rate >85% of predict also correlates with maximum physiological effort but is often not achieved in patients on beta blockers and/or with chronotropic incompetence.

In case maximal effort is achieved, peak $\dot{V}$O_2_ response is the gold standard metric of fitness. Peak $\dot{V}$O_2_ < 14 mL/kg/min predicts a poor prognosis [36,37], with some caveats; in patients on beta blockers, the lower threshold value is <12 mL/kg/min [46,47], and in young patients and patients with high or low BMIs, peak $\dot{V}$O_2_ should be interpreted as a percentage of the prediction, with values <50% indicating a poor prognosis [48]. The peak $\dot{V}$O_2_ can be used to risk-stratify the HF population in groups; Weber classes A, B, C, and D corresponding to peak $\dot{V}$O_2_ > 20, 16-to-20, 10-to-16, and <10 mL/kg/min were associated with a 3-year transplant and mechanical circulatory support-free survival of 97%, 93%, 83%, and 64%, respectively [49]. In patients who do not reach a maximum effort, analysis should consider O_2_ uptake variables that are independent of maximal effort; an oxygen uptake efficiency slope <1.4 and a $\dot{V}$O_2_ at AT <9 mL/kg/min indicate a poor prognosis [50].

### 7.3. CPET Studies in Patients with Heart Failure with Preserved Ejection Fractions

Nadruz et al. studied the clinical characteristics, CPET results, and outcomes in 195 patients with heart failure with preserved ejection fractions (>50%), 144 patients with heart failure with mid-range ejection fractions (40–49%), and 630 heart failure patients with reduced ejection fractions (<40%) [41]. There were significant differences in the clinical characteristics among these three groups. For example, patients with reduced ejection fractions had a higher prevalence of diabetes and coronary disease. Patients with a reduced ejection fraction had a mean peak $\dot{V}$O_2_ of 14.3 ± 5.2 mL/kg/min, patients with mid-range ejection fractions had a mean peak $\dot{V}$O_2_ of 17.1 ± 7.1 mL/kg/min, and patients with a preserved ejection fraction had a mean peak $\dot{V}$O_2_ of 17.4 ± 7.8 mL/kg/min. The $\dot{V}$E/$\dot{V}$CO_2_ slopes were 34.5 ± 1.2, 29.5 ± 6.3, and 30.3 ± 6.7 in these three groups, respectively. Patients with reduced ejection fractions have lower peak heart rates, lower peak systolic blood pressures, and lower peak diastolic blood pressures. In multivariable Cox models, including clinical predictors, the peak $\dot{V}$O_2_ and $\dot{V}$E/CO_2_ slope were both independently associated with composite outcomes in all three groups. These outcomes included all-cause death, left ventricular assist device implantation, and heart transplantation. Patients with two abnormal results in comparison to one abnormal result on CPET had an increased incidence of composite outcomes and heart failure hospitalizations in all three groups. In summary, CPET provides important information regarding outcomes in patients with heart failure in all three categories of ejection fractions, including patients with preserved ejection fractions.

Ho et al. analyzed clinical information, exercise testing results, and outcomes in 461 patients with left ventricular ejection fractions ≥50% and New York Heart Association Class II–IV symptoms [49,51]. These patients frequently had comorbid diseases, including hypertension, obesity, diabetes, and atrial fibrillation. The patients were placed in separate categories using criteria established by the American College of Cardiology/American Heart Association (ACC/AHA), the European Society of Cardiology (ESC), and the Heart Failure Society of America (HFSA). The use of three different criteria resulted in significant differences in the number of patients classified as HFpEF. The peak $\dot{V}$O_2_ max ranged from 16.2 ± 5.2 mL/kg/min in patients classified by ACC/AHA criteria to 12.7 ± 3.1 mL/kg/min in those classified by HFCA criteria. Patients in all three classification groups had elevated $\dot{V}$E/$\dot{V}$CO_2_ slopes. Two hundred forty-three patients underwent right heart catheterization, which provided pulmonary capillary wedge pressure measurements; 29%-to-63% of patients had elevated resting pulmonary capillary wedge pressures, and 16%-to-31% had abnormal exercise-induced pulmonary catheter wedge pressures, depending on the classification group. Differences in cardiovascular outcomes depended on the patients’ clinical classification, and they ranged from 75 events per 1000 person-years in ACC/AHA patients to 298 events per 1000 person-years in patients in HFSA classification. This study demonstrates that there are significant differences in clinical profiles, CPET results, and clinical outcomes in patients with HFpEF depending on the criteria used for classification. This heterogeneity in these patients complicates management and outcome studies.

Verwerft et al. analyzed the clinical characteristics, CPET results, subsequent testing recommendations, and treatment modifications in 297 patients with HFpEF referred to a dedicated dyspnea clinic [48]. These patients had impaired chronotropic reserve (72%) and impaired stroke volume reserve (73%). The overall impairment in cardiac output reserve was 59%, and 65% had pulmonary hypertension during exercise. Forty percent of patients had impaired O_2_ extraction in exercising muscle. One hundred and sixteen patients had a pulmonary disorder contributing to their exercise impairment. Most patients (267, 90%) were referred for additional diagnostic evaluation, with a median of two tests per patient. Nearly all patients (293, 99%) had changes in their cardiovascular medications. This study demonstrates that patients with HFpEF with unexplained dyspnea should have a comprehensive evaluation that includes both cardiac and pulmonary testing and routine and specialized laboratory studies. Many patients have more than one disorder, which contributes to their dyspnea.

Sachdev et al. published a scientific statement from the American Heart Association and the American College of Cardiology on supervised exercise testing for patients with HFpEF [52]. This document summarizes multiple sources of data that demonstrate that exercise training can improve peak $\dot{V}$O_2_, total exercise time, 6 min walk distance, quality of life, and muscle function. They reported a meta-analysis of six studies, which included 212 patients that demonstrated that the peak $\dot{V}$O_2_ increased from 15.7 to 17.7 mL/kg/min with exercise training. Consequently, this statement concludes that patients with HFpEF should routinely participate in supervised exercise training, and the benefits will likely exceed those associated with current drug therapy.

These studies demonstrate that patients with HFpEF can have complex clinical profiles with significant comorbidity, have abnormal exercise results on CPET, and have adverse outcomes, including all-cause mortality and hospitalization. They need a comprehensive evaluation, and they will benefit from supervised exercise training.

## 8. Cardiopulmonary Exercise Testing and Drug Trials

When patients are classified according to their peak $\dot{V}$O_2_, patients with a peak $\dot{V}$O_2_ less than 12 mL/kg/min had a reduced diffusion capacity, a reduced membrane component of diffusion, a reduced vital capacity, and a reduced alveolar volume. This indicates that pulmonary function tests can identify important changes in pulmonary function in patients with chronic HF and potentially help explain changes in their exercise capacity [51,53].

Patients treated with angiotensin-converting enzyme (ACE) inhibitors have a significant increase in lung diffusion, exercise tolerance, and peak $\dot{V}$O_2_, a decrease in peak dead space/tidal volume ratio, and a decrease in the $\dot{V}$E/$\dot{V}$CO_2_ slope. These results suggest that ACE inhibitors improve gas exchange at the alveolar capillary level, which is probably secondary to increases of prostaglandin concentrations. These results require at least 1 month of treatment. Aspirin therapy may reduce or limit the benefit associated with ACE inhibitors. Angiotensin II receptor blockers also increase exercise tolerance and peak $\dot{V}$O_2_ [51].

Studies with carvedilol indicate that it does not affect pulmonary mechanics or exercise capacity. Neither peak workload nor $\dot{V}$O_2_ improved with the drug. However, quality-of-life scores did improve, and there were reduced ventilatory responses to exercise, demonstrated by a decrease in the $\dot{V}$E/$\dot{V}$CO_2_ slope. Patients on carvedilol had a significant decrease in diffusion capacity, which was explained by a reduction in membrane conductance. The diffusion capacity on carvedilol was significantly lower than the diffusion capacity in patients treated with bisoprolol or nebivolol. Additional studies suggest that patients on carvedilol have a lower sensitivity to both CO_2_ and O_2_, and this reduces their ventilatory response during exercise [51].

Spironolactone appears to improve gas transfer in the lung and exercise capacity. This result may reflect some reversal in the damage in alveolar capillary membranes and a slowing of the development of interstitial fibrosis. In patients treated with an angiotensin receptor–neprilysin inhibitors, peak oxygen consumption increased, the $\dot{V}$E/$\dot{V}$CO_2_ slope decreased, $\dot{V}$O_2_ at the anaerobic threshold increased, and oxygen pulse increased in one study. However, Campanile et al. studied the addition of sacubitril/valsartan to standard medical therapy on the exercise capacity of patients with HFrEF [54]. This study included 12 patients on standard medical therapy plus sacubitril/valsartan and 13 patients on standard therapy. The median study time was 16 months. There was no significant change in the peak $\dot{V}$O_2_ at follow-up in comparison to baseline measurements. In addition, there was no significant change in the $\dot{V}$E/$\dot{V}$CO_2_ slope. The peak $\dot{V}$O_2_ was 12.2 ± 4.6 mL/kg/min at baseline and was 12.7 ± 3.3 mL/kg/min at follow-up. The mean $\dot{V}$E/$\dot{V}$CO_2_ slope was 35.4 ± 7.4 at baseline and was 37.2 ± 13.1 at follow-up. Patients in the control standard medical therapy group had nearly identical parameters at baseline and at follow-up. Consequently, after a median follow-up of 16 months, there was no significant change in CPET parameters with the addition of sacubitril/valsartan to standard medical therapy in patients with congestive heart failure with reduced ejection fractions. Sodium glucose co-transporter–2 inhibitors increased peak $\dot{V}$O_2_ and decreased $\dot{V}$E/$\dot{V}$CO_2_ slopes. Intravenous ferric carboxymaltose improves peak $\dot{V}$O_2_ [51].

These results indicate that patients with CHF should have pulmonary function tests, especially diffusion capacity measurements. It is impractical to use exercise testing to make drug choices in individual patients, but these studies provide the basis for clinical trials with drugs. The drugs discussed in this section all increased peak $\dot{V}$O_2_. Expert committees should summarize studies measuring CPET information on various drug regimens.

## 9. Biomarkers, Cardiopulmonary Exercise Testing, and Cardiac Rehabilitation

Kruger et al. prospectively studied 70 patients with chronic HF who underwent symptom-limited bicycle exercise testing [55]. The mean age of these patients was 60.3 ± 10.4; the mean ejection fraction was 26.4 ± 6.0%. There was a significant negative correlation between BNP concentration and peak $\dot{V}$O_2_, $\dot{V}$O_2_ at the anaerobic threshold, the relationship between minute ventilation and CO_2_ production, the number of Watts reached during exercise, and the left ventricular ejection fraction. BNP levels could discriminate between patients who had a peak $\dot{V}$O_2_ less than 10 mL/kg/min or a peak $\dot{V}$O_2_ less than 14 mL/kg/min. These authors suggest that BNP may be an effective way to monitor therapy and exercise programs in patients with HF.

Conraads studied the outcomes in 27 patients who participated in a combined exercise program that included both endurance training and resistance training for 4 months [56,57]. These patients were 59 ± 2 years of age and had a left ventricular ejection fraction of 26 ± 1%. After 4 months, there was a significant decrease in NT–proBNP levels, a decrease in New York Heart Association classification, an increase in $\dot{V}$O_2_ peak, an increase in $\dot{V}$O_2_ at the anaerobic threshold, and a decrease in the left ventricular end-systolic diameter. There was a significant negative correlation between peak $\dot{V}$O_2_ and NT–proBNP. These authors suggest that exercise training offers a nonpharmacologic method to modulate the active neurohormonal pathway in patients with HF.

Smart et al. carried out a patient-level meta-analysis of the effect of exercise training on BNP levels and NT-proBNP in patients with HF [58]. They identified 10 randomized controlled trials that measured BNP or NT–proBNP and obtained individual patient data on 565 patients from these trials. Exercise training reduced BNP by 28.3%, reduced NT–proBNP by 37%, and increased peak $\dot{V}$O_2_ by 17.8%. There is a significant negative correlation between the change in BNP and NT-proBNP and the change in peak $\dot{V}$O_2_. Changes in BNP and in NT–proBNP and the peak $\dot{V}$O_2_ were analyzed in the various subgroups of patients. Patients with a left ventricular ejection fraction less than 34% had statistically significant decreases in BNP and NT–proBNP with training. Patients with a peak $\dot{V}$O_2_ less than 14 mL/kg/min in the age category >60 had reductions in BNP and NT-proBNP levels, but these reductions did not reach the conventional level of statistical significance (*p* > 0.05). Overall, this study demonstrates that patients with HF and left ventricular ejection fractions of 35% have significant reductions in BNP and NT–proBNP following controlled trials of aerobic and/or resistance exercise training. Consequently, biomarkers can provide important information about changes in physical capacity and outcomes in rehabilitation programs.

MicroRNA (miRNAs) are non-coding RNAs that help regulate gene expression by inhibiting transcription. The interaction of miRNAs with their target genes depends on the intracellular location of miRNAs, the concentration of the miRNAs and target mRNAs, and the degree of binding of the miRNA to mRNA. These microRNA levels have been studied in patients with chronic HF participating in cardiac rehabilitation. Witvrouwen et al. compared plasma miRNA levels in 25 patients with heart failure enrolled in a 15-week combined aerobic and strength exercise program with 21 sedentary males to determine the effect of exercise [59]. These investigators measured miR-23a, miR-140, miR-146a, miR-191, and miR-210 levels, which are associated with pathways relevant to exercise adaptation, in both patient groups. The baseline miRNA expression was similar between groups except for miR-23a, which was higher in patients referred for cardiac rehabilitation. In this study, patients with lower LVEFs had higher miR-210 levels, independent of age. Baseline miR-23a was significantly associated with a percent change in $\dot{V}$O_2_ peak after completion of the rehabilitation program. MiR-146a decreased following 15 weeks of training. Therefore, these measurements indicate that miRNAs can help classify patients and measure outcomes during rehabilitation.

In HF patients with reduced ejection fractions, capillary density in skeletal muscle is reduced. Both miR-23 and miR-146a stimulate angiogenesis; therefore, reduced miR-23a and miR-146A levels after 15 weeks of training may reflect increased capillary density and a reduced need for angiogenesis. In addition, HF patients with reduced ejection fractions and reduced physical activity levels often have skeletal muscle wasting, and this contributes to fatigue during daily activities and reduces $\dot{V}$O_2_ peak during CPET. Both miR-23 and miR146a limit skeletal muscle atrophy by inhibiting the ubiquitin–proteasome pathway and Wnt family member 11 expression, and increased levels of these miRNAs suggest that the patient is having adaptive responses in skeletal muscle to prevent wasting. In conclusion, this study demonstrates that exercise effects the levels of miRNAs involved in pathways related to exercise adaptation, which may help improve skeletal muscle and the angiogenic response to exercise in HFrEF patients. This study also demonstrates that measuring miRNAs may help classify patients into phenotypes, monitor responses to treatment, especially exercise rehabilitation, and possibly understand the pathogenesis of muscle dysfunction in HF patients [59,60,61,62].

## 10. Limitations of CPET in Heart Failure

Cardiopulmonary exercise testing can clearly characterize the physical capacity of patients with heart disease. This testing can determine whether the predominant limitation involves the cardiac system, the respiratory system, or the neuromuscular system. Identification of the major impairment allows clinicians to focus on a particular system with either medications or exercise programs. There is a good correlation between peak $\dot{V}$O_2_ and outcomes. However, cardiopulmonary exercise testing requires additional equipment and trained personnel to undertake this testing safely. These exercise tests generate significant amounts of information, which require interpretation by specialists trained in exercise testing. These requirements generate increased costs for patient care and a time commitment for patients. In addition, many facilities do not have this equipment and cannot characterize their patients. An alternative approach is a 6 min walk test. However, this test has limitations related to the walk course, the level of exertion of the patient during the test, and the interaction between the patient and the personnel conducting the test [63].

## 11. Developing Technology

Portable metabolimeters can measure oxygen uptake during different physical activities and exercise protocols outside the cardiopulmonary lab [55]. This allows the clinician to relate oxygen uptake in activities, such as walking upstairs, with peak oxygen uptake measured during CPET. Patients with HF have higher $\dot{V}$O_2_ levels, reported as a percent peak $\dot{V}$O_2_, and higher $\dot{V}$E/$\dot{V}$CO_2_ slopes than healthy individuals in various routine activities of daily living. In addition, the times to complete tasks are longer in patients with HF. In patients with advanced HF ($\dot{V}$O_2_ < 12 mL/kg/min), the $\dot{V}$O_2_ for activities, such as making the bed, sweeping the floor, and walking upstairs, can represent up to 100% of their peak $\dot{V}$O_2_. Therefore, this portable equipment allows clinicians to measure O_2_ uptake requirements for specific activities in patients with HF who complain of dyspnea during these activities. This could be carried out during clinic evaluations with some activities, such as walking upstairs, or in rehabilitation programs, which create small rooms to carry out various activities of daily living. These results would provide both patients and clinicians with a better understanding of the patient’s symptom profile. In addition, these measurements could also be made during 6 min walk tests, which would increase the physiologic information available during this commonly used test and would provide a more complete analysis of disease progression and responses to changes in the patient’s medical regimen [63].

Recent technological advances have added significant capability to watches so that now smart watches can provide clinically relevant information to patients and their physicians [61,62]. These watches can make on-demand blood pressure measurements, determine heart rates and arrhythmias such as atrial fibrillation, measure O_2_ saturations, and measure sleep apnea parameters. This information can detect new problems and provide the basis for additional, more formal, testing. In addition, these devices can measure responses to treatment for hypertension and atrial fibrillation during routine daily activities. Patients could buy these devices or clinics could provide them for brief periods to answer specific questions. This technology will likely continue to advance, and healthcare systems will need to identify experts to keep up with these advances and understand the best application of particular devices for particular medical problems.

## 12. Potential for Personalized Medicine

**Figure 2:**
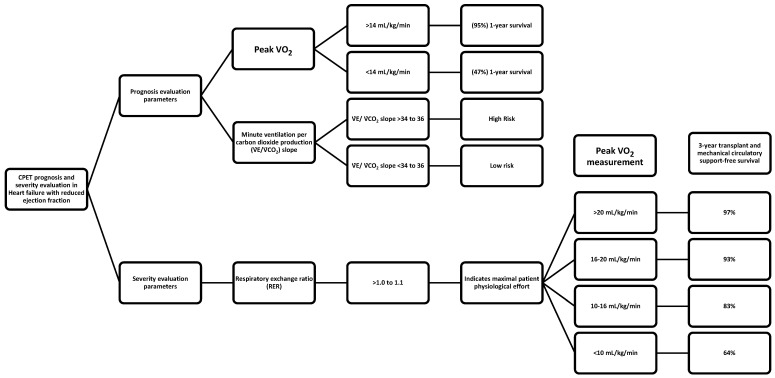
The use of CPET to classify patients and make estimates of outcomes.

Cardiopulmonary exercise testing provides important information about the physical capacity of patients with HF and usually can determine the limiting factor or factors in their exercise performance (Figure 2). This information becomes particularly important when respiratory disease or muscular disease make a substantial and perhaps unexpected contribution to this impairment. This should lead to additional evaluation to determine the type of respiratory or muscle disease and possible treatment options. If the exercise limitation largely reflects cardiac impairment, then the cardiopulmonary testing can classify patients according to their $\dot{V}$O_2_ max. This provides a prediction about longevity and the potential need for advanced treatment with heart transplantation, if available. However, other comorbidities may have important effects on cardiac dysfunction and disease progression, and in many cases personalized medicine requires the optimal management of all comorbidity and not just the addition of specific cardiac medications. In particular, careful management of hypertension and obstructive sleep apnea can reduce the progression of cardiac dysfunction and improve the quality of life. Recent advances, including the development of portable devices to measure O_2_ consumption, CO_2_ production, ventilation, O_2_ saturation, and heart rate and rhythm, provide relatively simple ways to monitor disease and make changes in treatment. Finally, smart watches provide patients with simple and inexpensive methods to monitor their health status and physical activity levels, and clinicians should encourage their use and should prescribe exercise programs based on the number of daily steps.

## 13. Conclusions

Cardiopulmonary exercise testing provides important information about the cardiac status of patients with HF. It measures the $\dot{V}$O_2_ max, which helps classify patients into categories with different outcome expectations. This testing helps clinicians understand patients’ symptoms and can help determine whether or not the respiratory system and the neuromuscular system contribute to patient limitations. In clinical trials, CPET can be used to determine changes in cardiac function and exercise capacity during drug treatment. However, cardiopulmonary testing requires specialized clinical laboratories, requires experts to interpret these tests, and is not available in most centers. Portable exercise units have the potential to provide onsite exercise studies in clinics, and these units can also help understand patients’ symptoms during routine activities. Finally, smart watches provide important information about patients during their daily routine activities. These devices can help detect undiagnosed medical problems and can help monitor responses to treatment. The organized and systematic use of smart watches could make important contributions to health care outcomes.

## Data Availability

All information in this manuscript is available in the reference list.
